# Functional Dimerization of Serotonin Receptors: Role in Health and Depressive Disorders

**DOI:** 10.3390/ijms242216416

**Published:** 2023-11-16

**Authors:** Elena V. Mitroshina, Ekaterina A. Marasanova, Maria V. Vedunova

**Affiliations:** 1 Institute of Biology and Biomedicine, Lobachevsky State University of Nizhny Novgorod, 23 Gagarin Avenue, 603022 Nizhny Novgorod, Russia; marasanova-2000-k@yandex.ru (E.A.M.);; 2 Faculty of Biology and Biotechnology, HSE University, St. Profsoyuznaya, 33, 117418 Moscow, Russia

**Keywords:** serotonin, 5-HT, 5-HT receptors, depression, receptor dimerization

## Abstract

Understanding the neurobiological underpinnings of depressive disorder constitutes a pressing challenge in the fields of psychiatry and neurobiology. Depression represents one of the most prevalent forms of mental and behavioral disorders globally. Alterations in dimerization capacity can influence the functional characteristics of serotonin receptors and may constitute a contributing factor to the onset of depressive disorders. The objective of this review is to consolidate the current understanding of interactions within the 5-HT receptor family and between 5-HT receptors and members of other receptor families. Furthermore, it aims to elucidate the role of such complexes in depressive disorders and delineate the mechanisms through which antidepressants exert their effects.

## 1. Introduction

Understanding the neurobiological underpinnings of depressive disorder constitutes a pressing challenge in the fields of psychiatry and neurobiology. Depression represents one of the most prevalent forms of mental and behavioral disorders globally. According to the World Health Organization, an estimated 3.8% of the global population grapples with depressive disorder, encompassing 5% of adults and 5.7% of adults aged 60 years or older. Notably, women are disproportionately affected by depressive disorders. Depression ranks as a leading cause of disability on a worldwide scale. Studies have illuminated that 15% of patients undergoing treatment for depression ultimately succumb to suicide, contributing significantly to a reduced life expectancy and elevated mortality risk associated with depression [1,2].

Clinically, depression manifests as an exceedingly heterogeneous disorder, influenced by an array of factors, spanning social, genetic, biological, and psychological dimensions [3]. The pathogenetic mechanisms underpinning depressive disorder, as well as the overarching comprehension of neurochemical systems linked to mood disorders, remain intricate and only partially elucidated. Depression is posited to stem from the dysfunction of multiple neurotransmitter systems, notably including the serotonergic system [4]. Serotonin (5-HT), a pivotal neurotransmitter, assumes a role in regulating sleep, learning, mood, and appetite. The efficacy of contemporary antidepressants is closely intertwined with their capacity to augment serotonin neurotransmission in some manner. Indeed, serotonin receptors, serving as the targets for serotonin, are also implicated in the pathogenesis of depressive disorders.

Moreover, serotonin, beyond its classical role as a neurotransmitter, plays a significant part in the regulation of neuronal development, encompassing processes like neurite outgrowth, somatic morphology regulation, axonal growth cone motility, and dendritic spine shape modulation [5,6]. There exists a hypothesis positing the involvement of several serotonin receptors (5-HTR), notably 5-HT1A and 5-HT2, in mediating the morphogenic effects induced by serotonin [7,8,9,10].

In recent years, a substantial body of literature has emerged, delving into the investigation of receptor interactions and functional dimerization in the brain, including serotonin receptors.

Studies have indicated the formation of homodimers among various subtypes of 5-HT receptors, including 5-HT1A [11], 5-HT2A [12], 5-HT1B [13], 5-HT2C [14], 5-HT4 [15], and 5-HT7 [16]. Additionally, evidence has surfaced in the past 5–7 years that suggests heterodimerization among different subtypes of 5-HT receptors (5-HT1B-5-HT1D [17], 5-HT1A-5-HT7 [18], 5-HT1A-5-HT2A [18], 5-HT2A-5-HT2B or with HT2A-5-HT2C and 5-HT2B-5-HT2C [19]. Furthermore, some 5-HT receptors engage in heterodimeric complexes with receptors outside the 5-HT receptor family, such as mu-opioid receptors [20] and dopamine D2 receptors [21], metabotropic glutamate receptors mGlu2 [22], cannabinoid receptors CB1 [23], and TrkB receptors [24].

Alterations in dimerization capacity can influence the functional characteristics of serotonin receptors and may constitute a contributing factor to the onset of depressive disorders. The objective of this review is to consolidate the current understanding of interactions within the 5-HT receptor family and between 5-HT receptors and members of other receptor families. Furthermore, it aims to elucidate the role of such complexes in depressive disorders and delineate the mechanisms through which antidepressants exert their effects.

## 2. The Role of Serotonin Receptors in the Pathogenesis of Depressive Disorders

Currently, there are at least 15 distinct subtypes of 5-HT receptors, categorized into seven classes or subfamilies [25]; these subtypes exhibit variations in structural and functional characteristics (Table 1). With the exception of serotonin type 3 (5-HT3) receptors, which function as ligand-gated ion channels, all serotonin receptor subtypes are part of the GPCR (G-protein-coupled receptor) superfamily [26]. Moreover, each receptor type may have different splicing and editing variants, can be modulated by auxiliary proteins and chaperones, and has the capability to form homo- or heterodimers [15,27]. This extensive diversity underscores the physiological significance of serotonin.

Given that these receptors are concurrently expressed in various tissue and cell types and bind with the same ligand (5-HT), studying the specific physiological roles of individual serotonin receptor subtypes is a formidable and ongoing task. 5-HT and its receptors are distributed throughout both the central and peripheral nervous systems, as well as in non-neuronal tissues of the intestine, cardiovascular system, and blood [26].

From an evolutionary perspective, 5-HT stands out as one of the most ancient neurotransmitters with a broad spectrum of roles in both physiological and pathological contexts. It is involved in regulating cognitive, behavioral, and emotional functions, as well as playing a crucial role in nervous system development and the establishment of synaptic connections [5,6,7,8,9,10]. Dysregulation of the serotonergic system has been implicated in the pathogenesis of various medical conditions, encompassing depression, anxiety, social phobia, schizophrenia, obsessive-compulsive and panic disorders, migraine, hypertension, pulmonary hypertension, eating disorders, vomiting, and more recently, irritable bowel syndrome [44].

**Figure 1:**
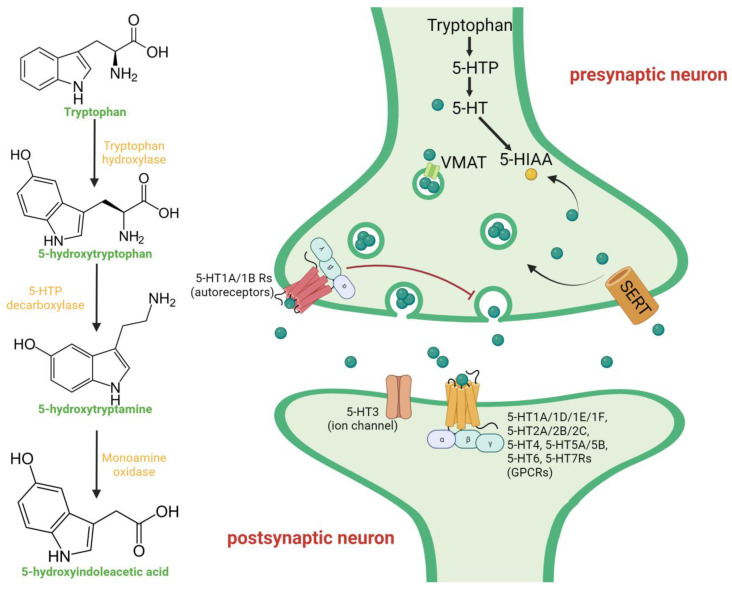
Scheme of the serotonergic transmission. Serotonin (5-hydroxytryptamine) is synthesized from tryptophan via 5-hydroxytryptophan with the participation of the tryptophan hydroxylase and 5-hydroxytryptophan decarboxylase. The neurotransmitter is packaged into presynaptic vesicles by VMAT and transported to the presynaptic membrane. After releasing into the synaptic cleft, serotonin can interact with 5-HT receptors on the postsynaptic membrane or with 5-HT autoreceptors on the presynaptic membrane. Interaction with autoreceptors is a negative feedback mechanism that prevents further release of serotonin. Serotonin receptors on the postsynaptic membrane include G-protein-coupled receptors and ligand-activated ion channels (5-HT3). Excess serotonin from the synaptic cleft can be uptaken to the presynaptic terminal by the serotonin transporter SERT. At the presynaptic terminal, serotonin can be repackaged into vesicles or deaminated to 5-hydroxyindoleacetic acid by monoamine oxidase. Figure was created with biorender.com.

In normal serotonergic neurotransmission, serotonin is synthesized from the amino acid tryptophan with the participation of the enzyme tryptophan hydroxylase and then transported from the cytoplasm of 5-HT neurons to the membrane via the H+-coupled vesicular monoamine transporter (VMAT) (Figure 1). VMAT is responsible for packaging monoamines into presynaptic vesicles. Subsequently, serotonin is released from the presynaptic terminal into the synaptic cleft, where it binds to postsynaptic 5-HT receptors, initiating signal transmission. Alternatively, it may bind to autoreceptors on the presynaptic membrane, which provide negative feedback and inhibit further serotonin release into the synaptic cleft. When there is an excess of serotonin in the synaptic cleft, it is reabsorbed by the highly selective serotonin transporter (SERT) located on the presynaptic membrane [45,46]. SERT transports free serotonin back into the neuron terminal, where it is repackaged into vesicles, thus repeating the cycle. Any serotonin that remains free in the cytoplasm and is not stored in vesicles is subject to deamination by monoamine oxidase in the mitochondrial membrane; this process results in the formation of the biologically inert metabolite 5-hydroxyindole-3-acetic acid (5-HIAA) [47].

G-protein-associated postsynaptic serotonin receptors play a role in regulating the activity of various adenylate cyclases and phospholipase C. Phospholipase C is responsible for triggering the synthesis of inositol triphosphate (IP3) and diacylglycerol (DAG), which in turn activate protein kinase C. Post-mortem brain studies on depressed individuals who died by suicide have utilized magnetic resonance spectroscopy to reveal a reduction in inositol content within the frontal cortex [48,49]. Cyclic adenosine monophosphate (cAMP), generated during the adenylate cyclase reaction, is an intracellular second messenger. It interacts with various targets, including protein kinase A and cyclic nucleotide-gated ion channels, leading to the modulation of calcium ion flow, changes in membrane excitability, and regulation of other cellular processes. Overall, this has a strong modulating effect on neuronal activity [27]. By activating protein kinase A, cAMP induces the phosphorylation of cAMP-sensitive transcription factors, such as the CREB protein (cAMP response element binding protein). When in its active form, pCREB promotes the expression of neurotrophic factor BDNF and various other proteins [50]. Protein kinase C also has an effect on CREB [51]. Expression levels of PKA, PKC, and adenylate cyclase were notably reduced in postmortem brains from individuals who died by suicide or suffered from major depression [52,53]. This decrease was also observed in the brains of depressed animals, implying disruptions in the serotonin system [54]. Intriguingly, the chemical PKA activator 8-bromo-cAMP demonstrated antidepressant activity [55]. Conversely, inhibitors of PKA and PKC blocked the effects of antidepressants [56,57].

Research has shown that the administration of nearly all antidepressants increases CREB and pCREB levels in the hippocampus and cortex [58]. Moreover, enhancing CREB expression in the dentate gyrus through gene transfer using viral vectors (AAV) mediates antidepressant-like effects in behavioral depression models, such as learned helplessness and forced swim tests [59].

ERK (extracellular signal-regulated kinase) is an intermediate component in the MAPK cascade responsible for activating the transcription factor CREB. ERK is activated, among others, by protein kinases A and C [60,61]. The Ras-Raf-MEK1/2 pathway is responsible for ERK activation through MEK-mediated dual threonine and tyrosine phosphorylation of ERK. In contrast, dual-specificity MAPK phosphatase (MKP) and serine/threonine protein phosphatase (PP) dephosphorylate and inactivate ERK [62]. In both humans and various animal models of chronic depression, ERK signaling was significantly reduced in the prefrontal cortex and hippocampus—two major regions implicated in depression [63]. Inhibiting the ERK pathway in these regions induced depressive-like behavior. Various antidepressants partially exerted their behavioral effects by normalizing decreased ERK activity [60].

The ERK pathway can also be activated independently of PKA through the interaction of cAMP with the Epac1/2 family of cAMP sensors (exchange proteins directly activated by cAMP). Two isoforms of Epac exist: Epac1 is expressed in embryonic nerve cells, while Epac2 is found in the amygdala and hippocampus of the adult brain. These proteins activate small GTPases Rap1/2, which, in turn, induce the phosphorylation of ERK kinase and its subsequent activation, leading to CREB phosphorylation [7,27]. Experimental evidence supports the involvement of this molecular cascade in mediating depressive effects. Rap expression was reduced in individuals who died by suicide and suffered from depression. Interestingly, Epac2 expression was not reduced; instead, it increased in the prefrontal cortex and hippocampus. This may be explained by a compensatory reaction of Epac2 overexpression in response to reduced Rap GTPase expression (Dwivedi et al., 2006 [63]). Knockout mice lacking Epac2 (Epac2−/−) exhibited anxiety, anhedonia, and depression phenotypes in various behavioral tests, including the open field test, forced swim test, and sucrose preference test [64].

Accumulating evidence suggests that at least five serotonin (5-HT) receptor subtypes (5-HT1A, 5-HT1B, 5-HT4, 5-HT6, and 5-HT7) are involved in the pathogenesis of depressive disorder [65]. Some of these receptors (5-HT1A, 5-HT1b, 5-HT4, and 5-HT7) are known to form functional homo- and heterodimers. In what follows, we will delve deeper into the details of these receptors.

### 2.1. 5-HT1A and 5-HT1B Receptors

#### 2.1.1. 5-HT1A

The 5-HT1A receptor has been extensively studied within the serotonin receptor family. It is widely distributed throughout the brain, with particularly high densities found in limbic areas, the hippocampus, septal and cortical regions, as well as in the dorsal and median raphe nuclei of the midbrain [66].

Activation of somatodendritic 5-HT1A autoreceptors located on serotonergic neurons by endogenous serotonin plays an important role in the physiological control of their activity. During wakefulness, 5-HT neurons generate regular impulses at a low frequency. However, during periods of excessive stimulation, such as stress, there is an increased release of serotonin near the cell bodies of these neurons. This released serotonin then activates 5-HT1A autoreceptors, which help maintain low and regular neuronal activity. This process can be considered a form of negative feedback physiological “safety valves” to maintain homeostasis [67].

Postsynaptic 5-HT1A heteroreceptors are expressed in the populations of non-serotonin neurons, primarily within the limbic system, including the bodies and dendrites of glutamatergic neurons [68] or axons of GABAergic [69] and cholinergic neurons [70]. These receptors play a role in regulating the release of various neurotransmitters, including acetylcholine in the medial septum, glutamate in the prefrontal cortex, and dopamine in the ventral tegmental area [67].

Moreover, distinctions in the interaction between 5-HT1A autoreceptors and heteroreceptors with G-proteins have been documented. Autoreceptors are primarily associated with Gαi3, whereas heteroreceptors are mainly linked to Gαo in the hippocampus and exhibit an equal association with Gαo and Gαi3 in the cortex [71].

Considerable experimental evidence suggests a connection between depressive disorders and alterations in the expression and function of the 5-HT1A receptor in the brain, although findings from such studies often diverge [72]. For instance, an increase in the number of 5-HT1A autoreceptors has been reported in the postmortem brain of individuals who died by suicide due to depression [73] and in cases of bipolar depression [74], patients with depression [75] and in in vivo experiments in the paraventricular nucleus [76] and hippocampus [77] in rats under CUMS.

Conversely, a review by Shrestha et al. [78] reported that among the eight studies they reviewed on 5-HT1A expression in the brains of patients with MDD, four demonstrated a decrease in 5-HT1A receptor density, two showed no change, and only two indicated an increase in 5-HT1A receptor density. Furthermore, Bartlett et al. [79] found no correlation between the severity of a depressive episode and an increase in 5-HT1AR binding potential. Another study indicated a decrease in the expression of 5-HT1A in the reserpine model of depression in rats [80].

Polymorphisms in the 5-HT1A promoter (G/C (SNP) rs6295, HTR1A+272GG and others) have been associated with the development of major depressive disorder, suicide risk, and sensitivity to antidepressants [81,82,83]. They have also been linked to the risk of developing schizophrenia [84].

However, studies investigating the effects of 5-HT1AR activation and inhibition on depressive-like disorders have produced conflicting results, demonstrating that both stimulation and blockade of 5-HT1A receptors can yield antidepressant effects [85].

On the contrary, in various studies, the 5-HT1A receptor antagonist WAY-100635 attenuated the antidepressant effects of different drugs [86,87,88,89,90]. An increase in depressive symptoms in rodents when using the antagonist WAY-100635 has also been observed in the study by Xiao et al. [91]. Concurrently, the activation of postsynaptic 5-HT1A receptors with F15599, a biased 5-HT1A agonist, or the non-selective agonist F13714 has elicited antidepressant-like effects [87,92,93].

This divergence may be attributed to functional distinctions between 5-HT1A autoreceptors and heteroreceptors. While 5-HT1A is expressed both as a presynaptic autoreceptor in serotonergic neurons of the raphe nuclei [68] and as a postsynaptic receptor in various brain regions, including the hippocampus and cerebral cortex [94], these receptors exhibit distinct responses to stimulation. Specifically, chronic stimulation of 5-HT1A receptors has been demonstrated to lead to functional desensitization, but this effect is observed exclusively in presynaptic 5-HT1A autoreceptors [95,96].

#### 2.1.2. 5-HT1B

Similar to 5-HT1A receptors, 5-HT1B receptors are divided into autoreceptors located on serotonin neurons and heteroreceptors. While 5-HT1A receptors are abundant in the hippocampus and cortex, 5-HT1B receptors are predominantly found in the basal ganglia [97]. Additionally, they differ in their subcellular localization, with 5-HT1A receptors being somatodendritic and 5-HT1B receptors being located at axon terminals [68]. 5-HT1B receptors inhibit voltage-gated calcium channels at presynaptic terminals, leading to decreased neurotransmitter release [98]. Activation of the 5-HT1B receptor also increases the reuptake of serotonin [99].

5-HT1B receptors play a role in regulating aggressive behavior [100] and are implicated in the development of depression. Their activation reduces serotonin levels in the brain due to their influence on serotonin release, synthesis, and reuptake [99,101]. Studies have shown a decrease in 5-HT1B receptor function in patients with major depressive disorder (MDD) [102], and these patients tend to be less sensitive to 5-HT1B agonists, indicating reduced expression or desensitization of 5-HT1B receptors [103]. However, 5-HT1B mRNA expression is increased in the raphe nuclei of rat brains after stress and depression simulations. Overexpression of 5-HT1B receptors in the raphe by Herpes Simplex Virus leads to the development of depressive-like behavior after stress [104]. In rats, a decrease in 5-HT1B receptor mRNA in the raphe, but not in the cortex and hippocampus, occurs after selective serotonin reuptake inhibitor (SSRI) treatment [105,106]. This effect appears to be specific to 5-HT1B autoreceptors. PET imaging has shown that, in patients who have undergone effective cognitive-behavioral therapy for depression, 5-HT1B receptor binding is reduced in the brainstem [107].

A number of studies show that 5-HT1B receptor agonists have antidepressant effects in humans [108,109,110], and in rodents [111,112]. One of the possible mechanisms of this action is a reduction in Ca^2+^ entry into the presynapse through voltage-gated Ca^2+^ channels mediated by 5-HT1B heteroreceptors and a decrease in glutamate release due to a decrease in AC/cAMP/PKA activation [113]. However, genetic knockout of the receptor has also been shown to lead to antidepressant-like behavior and increased serotonin levels in mice [114,115,116], but these data were not confirmed in later studies.

These contradictory physiological functions of the 5-HT1A and 5-HT1B receptor subpopulations make them a complex and controversial target in the development of new effective antidepressants.

### 2.2. 5-HT4 Receptors

The 5-HT4 receptors are widely expressed in limbic areas such as the amygdala, septum, hippocampus, olfactory tubercle, frontal cortex, and basal ganglia, including the striatum and substantia nigra [27,117]. These receptors are primarily considered to be postsynaptic, although evidence suggests their presynaptic localization at the terminals of GABAergic (dentate gyrus), dopaminergic, and serotonergic neurons, since the release of these neurotransmitters is modulated by 5-HT4 agonists [33,118].

In addition to the brain, 5-HT4 receptors are found in the heart [119], intestines [120], adrenal glands [121], bladder [122], and cells of the immune system [123].

Similar to 5-HT1A receptors, 5-HT4 receptors in the prefrontal cortex influence the firing rate of midbrain serotonergic cells but exhibit an opposite modulatory effect, increasing the likelihood of action potential generation through the positive prefrontal cortex-dorsal raphe nucleus (PFC-DRN) feedback loop [124,125]. Administration of 5-HT4 receptor agonists has an excitatory impact on the activation of midbrain 5-HT cells [124,126]. Additionally, 5-HT4 receptors play a role in finely regulating fundamental cellular processes underlying synaptic plasticity, long-term potentiation (LTP) and long-term depression (LTD), which likely contribute to their effects on hippocampal-dependent long-term memory. 5-HT4 receptor activation prevents LTD in all major hippocampal subfields, and the effects of 5-HT4 activation on LTD and LTP vary across hippocampal fields [127].

Numerous studies have implicated 5-HT4 receptors in mood disorders. Notably, the C-terminal domain of these receptors displays diversity due to alternative mRNA splicing, and various variants have been associated with susceptibility to unipolar depression [128]. Changes in the expression of 5-HT4 receptors have been reported in various rodent models of depression, although these changes appear to vary depending on the specific model employed. Some studies have reported decreased 5-HT4 expression [129,130], while others have observed an increase [131]. In human studies, conflicting results have also been reported. For instance, increased 5-HT4 receptor binding and cAMP concentrations have been observed in the hippocampus of depressed suicide victims [132]. Conversely, a study by Madsen et al. [133] found a link between depression and reduced levels of 5-HT4 receptors in the caudate-putamen.

Certain studies have linked decreased serotonergic neuron activity in various brain regions and 5-HT4 receptor knockout to depressive disorders and anxiety-like behavior [124,125,134,135,136].

5-HT4 receptors play a significant role in the molecular mechanisms of action of antidepressants, and their agonists have demonstrated potential as effective antidepressants with a notably rapid onset of therapeutic effects compared to currently utilized antidepressants [137,138]. For instance, 5-HT4R agonists such as RS67333 and SL65.0155 have been found to increase the expression of BDNF mRNA in the hippocampus. This increased BDNF expression is likely a result of the additional activation of the CREB pathway, which subsequently stimulates the expression of various neurotrophic factors, including BDNF [139].

Furthermore, 5-HT4 receptors are implicated in adult neurogenesis since 5-HT4R agonists have been shown to enhance neurogenesis in the dentate gyrus and enteric nervous system. Conversely [137,139], 5-HT4R antagonists reduce the differentiation of neural progenitor cells (NPCs) with minimal impact on cell proliferation, maturation, or morphology [140]. Notably, mice deficient in 5-HT4 receptors exhibit a diminished neurogenic response to chronic SSRI treatment [141], and the phenomenon of dedifferentiation is less pronounced in these 5-HT4R-deficient mice [142].

### 2.3. 5-HT7 Receptors

5-HT7 receptors are the last subtype of serotonin receptors to be discovered; they were cloned in 1993 [143]. These receptors are characterized by their high expression levels in various functionally significant brain regions, including the thalamus, hypothalamus, hippocampus, prefrontal cortex, striatal complex, amygdala, and Purkinje neurons of the cerebellum [144]. In the amygdala, 5-HT7 receptors are initially found on GABAergic interneurons, while their expression in specific neurons in other brain structures is less clear [145]. The 5-HT7 receptor plays a role in regulating essential physiological processes such as circadian rhythms, the sleep–wake cycle, thermoregulation, learning and memory formation, and nociception [146]. Additionally, the expression of 5-HT7 has been identified in spinal cord [147], liver [148], immune system cells [123,149], heart, and kidneys [150].

The 5-HT7 receptor is implicated in the pathogenesis of a wide range of neurological disorders, including autism spectrum disorders (ASD), cognitive and affective dysfunctions, schizophrenia, depression, anxiety, impulsivity, epilepsy, migraine, and neuropathic pain [38].

**Figure 2:**
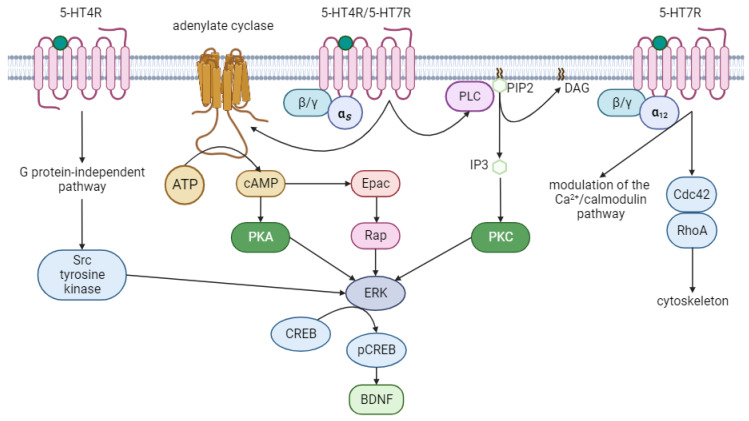
Signaling pathways activated by 5-HT4Rs and 5-HT7Rs. 5-HT4 and 5-HT7 are Gαs-coupled metabotropic receptors. They activate adenylate cyclase and phospholipase C. Activation of adenylate cyclase leads to the synthesis of an intracellular second messenger—cAMP. cAMP activates protein kinase A as well as cAMP Epac sensors. Protein kinase A and Epac activate ERK kinase, while activation through Epac occurs PKA-independently, through the activation of small GTPases Rap. Activated ERK is able to phosphorylate and thereby activate CREB. CREB is a transcription factor, which induces the expression of a number of neurotrophic proteins, in particular BDNF. Phospholipase C, in turn, cleaves membrane phosphatidylinositol biphosphate to form diacylglycerol and inositol triphosphate. Inositol triphosphate activates protein kinase C, which also leads to activation of ERK kinase. 5-HT4Rs are capable of G-protein independent signaling, namely they are able to directly activate Src tyrosine kinase, which will activate ERK kinase. 5-HT7Rs are capable of signaling through Ga12, which triggers the stimulation of Rho GTPases, Cdc42, and RhoA. These proteins are involved in the regulation of cytoskeletal organization. In addition, 5-HT7Rs modulate the Ca^2+^/calmodulin pathway. Green dots—serotonin or any selective agonist of corresponding receptor. Figure was created with biorender.com.

Figure 2 shows main signaling pathways activated by 5-HT4Rs and 5-HT7Rs.

Hippocampal 5-HT7 receptors appear to be involved in interactions between the serotonergic system and the hypothalamic-pituitary-adrenal (HPA) axis since 5-HT7R agonists have been shown to increase glucocorticoid receptor expression in hippocampal cell cultures [151]. Additionally, acute, but not chronic, immobilization stress increases 5-HT7R mRNA concentrations in the CA2 and CA3 regions of the rat hippocampus [152]. Administration of antidepressants has been found to suppress 5-HT7 expression in the hypothalamus [153]. Interestingly, 5-HT7 receptor knockout mice exhibit an antidepressant-like behavioral phenotype in the forced swim test [154,155]. They also exhibit antidepressant-like behavior under stressful conditions, and a pharmacological blockade of 5-HT7Rs results in a more rapid antidepressant response in rats [155,156]. The atypical antipsychotic amisulpride, which acts as a high-affinity 5-HT7R antagonist, also exerts antidepressant effects. Notably, these antidepressant-like behavioral effects of amisulpride are abolished in mice lacking 5-HT7Rs [157].

Selective blockade of 5-HT7 receptors (5-HT7R) has consistently demonstrated antidepressant-like effects in classical behavioral tests performed on laboratory rodents, including the tail suspension test and the forced swim test [156,158,159,160,161,162].

A recent study has shown that acute activation of 5-HT7R induces depressive-like behavior in mice, and this effect is mediated by matrix metalloproteinase MMP-9. It was also observed that the activation of 5-HT7R and chronic stress leads to the remodeling of dendritic spines, resulting in increased spine length but reduced dendritic density [163].

In vitro studies have indicated that the 5-HT7R agonist LP12 increases the expression of the BDNF receptor TrkB (tropomyosin receptor kinase B). This effect is likely mediated through cAMP-activated cascades since the activation of 5-HT7 receptors leads to increased adenylate cyclase activity, which, in turn, promotes TrkB expression [164]. The therapeutic activity of 5-HT7R antagonists is likely attributed to their presence on GABAergic neurons. Activation of 5-HT7 receptors triggers the release of GABA, which inhibits serotonergic activity and serotonin release [165,166].

Nevertheless, research involving pharmacological and genetic manipulations of 5-HT7R in animal models of depression and anxiety frequently yields conflicting outcomes. For instance, a study by Maxwell et al. [167] investigating two selective 5-HT7 receptor antagonists, DR-4004 and SB-269970, failed to show a significant antidepressant effect. Similarly, in research by Balcer et al. [154], the behavioral effects observed in 5-HT7 knockout animals did not consistently indicate the development of depressive disorders. Furthermore, in the study by Bonaventure et al., the initially demonstrated antidepressant effect of the 5-HT7R antagonist JNJ-18038683 was not substantiated in a subsequent clinical trial involving 225 patients with major depressive disorder [168].

In summary, serotonin receptors are undeniably linked to the development of anxiety and depressive conditions and play a role in the response to antidepressant therapy. Nonetheless, their effects are intricate and frequently contradictory. These discrepancies may be attributed to variations in experimental design, such as the choice of animal strain, specific behavioral tests, drugs used, drug dosages, and routes of administration. The use of nonselective drugs that affect multiple receptor subtypes can also contribute to these complexities. Consideration must be given to the variations in expression levels and functional actions of pre- and postsynaptic serotonin receptors. Presynaptic receptors play a pivotal role as primary regulators of serotonin neuron firing patterns and 5-HT release [169,170,171]. Typically, the stimulation of 5-HT1A and 5-HT1B autoreceptors exerts anxiolytic and antidepressant effects on behavior [171].

Furthermore, it is essential to consider the vast phenotypic diversity of serotonergic neurons. In an extensive study by Okaty et al. [172], a detailed transcriptomic atlas of Pet1-lineage neurons (transcription factor PET1 determines neuronal differentiation into serotonergic) of the dorsal raphe nucleus was established at cellular resolution. The authors identified 14 distinct clusters of serotonergic neurons, each expressing a unique repertoire of neurotransmitters, plasma membrane receptors, ion channels, cell adhesion molecules, and other gene categories. Such a comprehensive approach is crucial for elucidating the functions of neurons.

In recent years, an additional mechanism has been discovered through which serotonin receptors participate in the pathogenesis of anxiety and depressive disorders, namely, alterations in the level of functional oligomerization.

## 3. Functional Dimerization of Receptors

The potential for altering the functional activity of serotonin receptors is suggested to involve their dimerization. Previously, it was commonly believed that GPCRs exist and function as monomers that interact with their corresponding G-proteins with a 1:1 stoichiometry. However, over the past two decades, emerging biochemical, structural, and functional data have indicated that GPCRs can form oligomers [173,174]. This oligomerization can occur between receptors of the same type (homomerization) or between different subtypes of receptors within the same GPCR family or across different GPCR families (heteromerization). Oligomerization can specifically modulate receptor properties, altering their pharmacology by affecting ligand binding on individual protomers or creating new binding sites [174,175,176].

The earliest evidence supporting the presence of 5-HT receptor dimers/oligomers comes from co-immunoprecipitation (Co-IP) and Western blot studies involving receptor subtypes 1A, 1B, and 1D [13,17]. Research has demonstrated the ability of the 5-HT1B and 5-HT1D receptors to form homodimers when expressed separately and heterodimers when co-expressed [13]. Over the years, the combined use of Co-IP and FRET microscopy has provided substantial evidence supporting homodimerization of receptor subtypes 1A, 1B, 1D, 2A, 2C, 4, and 7 [11,14,15,177,178].

Further indications of the potential heterodimerization of different 5-HT receptor subtypes have been observed for combinations such as 5-HT1A with 5-HT7 [173], 5-HT1A with 5-HT2A [179], 5-HT2A with 5-HT2B or 5-HT2C, and 5-HT2B with 5-HT2C [19] receptors.

### 3.1. Homodimerization of Serotonin Receptors

**Figure 3:**
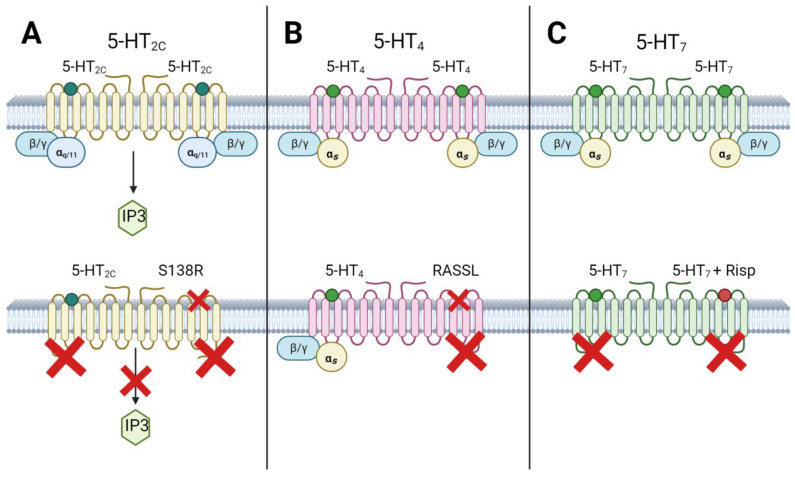
Homodimerization of serotonin receptors. (**A**) In the case of the formation of a 5-HT2C homodimer upon inactivation of one of the protomers, a complete blockade of signaling from the homodimer occurs, which leads to the inability of both protomers to activate G-proteins and stimulate the production of inositol triphosphate upon binding to 5-HT. (**B**) In the 5-HT4 homodimer, each protomer makes an equal contribution to the signal transduction process. Thus, inactivation of one of the protomers leads to a twofold decrease in the efficiency of G-protein activation compared to a homodimer with two intact protomers. (**C**) Inhibition of one of the protomers 5-HT7 homodimer by risperidone results in the entire dimer becoming inactive for the G-protein. Green dots—serotonin or any selective agonist of corresponding receptor; red dot—antagonist of 5-HT7R risperidone; red crossing—inability to bind ligand/activate G-protein/impossibility of signal transmission. Figure was created with biorender.com.

The functional significance of dimerization, particularly in terms of G-protein activation by serotonin receptors, has been studied for the 5-HT2C, 5-HT4, and 5-HT7 receptors (Figure 3). Regarding the 5-HT2C receptor, researchers created a mutant (S138R) receptor that could not bind to 5-HT, resulting in the loss of basal activity and the inability to activate G-proteins [180]. When wild-type (WT) receptors were co-expressed with the S138R mutant receptor, heterodimers formed, comprising one active and one inactive protomer with respect to G-protein signaling. Importantly, the WT-S138R heterodimer was incapable of activating G-proteins or stimulating inositol phosphate production in response to 5-HT stimulation [180]. This finding suggested that both protomers are involved in G-protein signaling, and signaling does not occur if one protomer remains in an inactive conformation. Furthermore, the dimerization of 5-HT2C receptors appears to be influenced by the presence of cholesterol in the cell membrane [181].

It is proposed that receptor dimerization may be a prerequisite for normal receptor trafficking and expression at the plasma membrane since it might be necessary to pass endoplasmic reticulum (ER) quality control checkpoints, which determine functionality. Additionally, it is possible that dimerization within the ER is necessary for transport to the plasma membrane, as dimers could represent a minimal structural and functional signaling unit [182]. Experimental evidence suggests that the dimerization of 5-HT2C receptors occurs before they reach the plasma membrane. Furthermore, co-localization of 5-HT2C receptor dimers with the ER, Golgi apparatus, and subsequently the plasma membrane has been observed [183].

The formation of disulfide bridges between 5-HT2A receptor’s transmembrane domain-3 and extracellular loop-2, plays a significant role in the establishment of homodimers [184,185]. Regarding 5-HT1A receptors, investigations have revealed that the extent of homodimer formation can be modulated by specific ligands. For instance, the agonist 8-OH-DPAT has been observed to enhance homodimerization of 5-HT1A receptor, whereas the antagonist methysergide diminishes it [178,186].

Studies conducted by Iglesias et al. have demonstrated that the minimal functional unit required for activation of the PLA2 and PLC pathways by the serotonin 5-HT2A receptor is the homodimeric complex [187].

Similar findings were ascertained for the 5-HT7 receptor, employing the antagonist risperidone, which pseudo-irreversibly binds to one protomer of the dimer [16]. These experiments have illuminated that irreversible binding of risperidone to one protomer of the dimer does not impede 5-HT from binding to the second protomer, yet it incapacitates the dimer with respect to G-protein activation. Investigations involving the 5-HT2C and 5-HT7 receptors substantiate the proposition that both protomers of the 5-HT receptor dimer partake in G-protein-mediated signaling. In cases where one protomer is restrained in an inactive conformation, either through mutagenesis or irrevocable occupation by an antagonist, the second protomer, though still proficient in binding 5-HT, cannot incite G-protein activation in response to 5-HT binding.

Furthermore, it has been elucidated that the 5-HT4 receptor exists as a constitutive dimer, which is probably formed with the involvement of disulfide bonds [188]. Studies employing ligand binding selective mutant 5-HT4-RASSL receptors have shown that the activation of a G-protein can be accomplished when an agonist binds to one protomer of the 5-HT4 receptor dimer. However, the efficiency of G-protein binding is twice as pronounced when both dimer protomers are activated via agonist binding [177]. This twofold augmentation in activation implies that binding to one protomer yields half-maximal activation, signifying an equitable contribution from each protomer to the signal transduction process [189].

Presently, there is a lack of conclusive evidence supporting the existence of homodimers in vivo for most GPCRs, including serotonin receptors. Nonetheless, investigations into the pseudo-irreversible binding of 5-HT7 and 5-HT2A receptor antagonists have revealed a noteworthy phenomenon: the reactivation of receptors initially inactivated by the antagonist when treated with alternative competitive antagonists. This phenomenon is attributed to allosteric crosstalk between protomers and may serve as indirect evidence suggesting the existence of dimers within native brain structures [190].

### 3.2. Heterodimerization between Different Serotonin Receptor Subtypes

**Figure 4:**
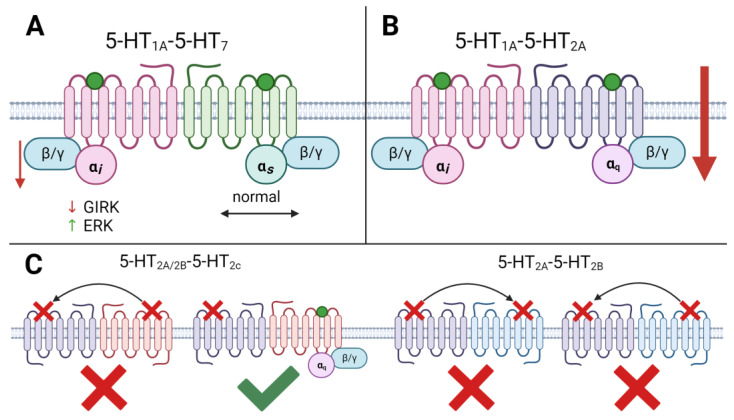
Heterodimerization between different families and subtypes of serotonin receptors. (**A**) Heterodimerization of 5-HT1A and 5-HT7 receptors leads to a decrease in the ability of the 5-HT1A receptor to activate the Gi protein, without affecting signal transmission by 5-HT7 receptors. In addition, heterodimerization reduces the ability of the 5-HT1A receptor to activate G-protein-dependent potassium channels, and also enhances the ability of the 5-HT1A receptor to activate MAPKs, particularly ERK. (**B**) Upon heterodimerization of 5-HT1A and 5-HT2A receptors, activation of the 5-HT2A protomer inhibits the ability of the 5-HT1A protomer to bind ligands. Thus, the 5-HT2A protomer has a dominant effect in the heterodimer. (**C**) Upon heterodimerization between subtypes of the 5-HT2 receptor family, the 5-HT2C protomer has a dominant effect in the 5-HT2A-5-HT2C and 5-HT2B-5-HT2C heterodimers. Blockade of the 5-HT2C protomer also blocks signal transmission from the 5-HT2A and 5-HT2B protomers. When blocking the 5-HT2A or 5-HT2B protomer, there is no blockade of the 5-HT2C protomer. In the case of the formation of the 5-HT2A-5-HT2B heterodimer, signal transmission from both protomers can be blocked if only one of the 5-HT2A or 5-HT2B protomers is blocked. Green dots—serotonin or any selective agonist of corresponding receptor; red crossing—inability to bind ligand/activate G-protein/impossibility of signal transmission; green checkmark—normal signal transmission. ↑—activation or increase releasing; ↓—inhibition or decrease releasing. Figure was created with biorender.com.

Heterodimerization presents a novel avenue for the differential regulation of signaling pathways, either by augmenting or inhibiting the original pathways established by corresponding homodimers, or by initiating entirely new pathways. Among 5-HT receptor subtypes, the 5-HT1A-5-HT7 heterodimer has garnered significant attention [18]. Serotonin receptors 5-HT1A and 5-HT7 are extensively co-expressed within brain regions implicated in depressive processes. The 5-HT1A-5-HT7 receptor heterodimers exhibit distinct functional attributes when contrasted with their homodimeric counterparts. Functionally, the process of heterodimerization leads to a reduction in 5-HT1A receptor-mediated activation of the Gi-protein, while leaving 5-HT7 receptor-mediated signaling unaffected. Furthermore, heterodimerization significantly diminishes the capacity of the 5-HT1A receptor to stimulate G-protein-dependent potassium channels (GIRKs) within a heterologous system. This inhibitory effect on GIRK channels remains evident within hippocampal neurons, underscoring the physiological relevance of heteromerization in vivo. Additionally, heterodimerization plays a pivotal role in the initiation of serotonin-mediated internalization of the 5-HT1A receptor, and it amplifies the receptor’s proficiency in activating mitogen-activated protein kinases (Figure 4) [18,173]. The evidence from Renner et al.’s work [173] strongly indicates a probable difference in the ratio of homo- and heterodimers formed in pre- and postsynaptic neurons. Specifically, the study suggests a higher prevalence of heterodimers in presynaptic neurons compared to postsynaptic neurons. This discrepancy likely serves as an explanation for the observed differential desensitization patterns between 5-HT1A auto- and heteroreceptors. Furthermore, research findings point out that the level of expression of postsynaptic 5-HT7 receptors in both the hippocampus [173] and forebrain [191] gradually decreases over the course of ontogenesis. In contrast, the expression of 5-HT1A receptors remains unchanged [173]. Taking these observations into consideration, it is plausible to infer that, under physiological conditions in adulthood, the abundance of 5-HT1A–5-HT1A homodimers is notably higher than that of 5-HT1A-5-HT7 heterodimers. This difference offers an explanation for the observed variations in the internalization processes of pre- and postsynaptic -HT1A receptors. Furthermore, the impact of heterodimerization extends to the 5-HT1A receptor-mediated activation of G-protein-gated potassium channels (GIRK), which is orchestrated by the Gβγ subunits of G-proteins [192].

Heterodimerization between 5-HT7 and 5-HT1A receptors potentially plays a role in the pathogenesis of depression. It has been suggested that an alteration in the balance between 5-HT1A/5-HT7 heterodimers and 5-HT1A/5-HT1A homodimers in presynaptic neurons may contribute to depressive states. Consequently, artificially increasing the number of 5-HT7 receptors in presynaptic terminals through the use of AAV viral vectors demonstrated antidepressant effects in both male mice of the C57Bl/6J reference line and ASC (antidepressant-sensitive catalepsy) mice genetically predisposed to depressive-like behavior. This effect is attributed to a shift in the ratio of 5-HT1A/5-HT1A homodimers to 5-HT7/5-HT1A heterodimers toward favoring heterodimerization [18,193]. In vivo experiments have provided additional insights, demonstrating a substantial reduction in the number of 5-HT1AR/5-HT7R heterodimers in the medial prefrontal cortex of rodents. This reduction correlates with depressive-like behavior induced by chronic unpredictable stress [194]. Furthermore, variations in the relative concentration of 5-HT1A-5-HT7 heterodimers in the raphe and hippocampus may account for regional disparities in 5-HT1A receptor binding to G-proteins [18].

Heterodimerization is also observed among the 5-HT2A, 5-HT2C, and 5-HT2B receptors when co-expressed in heterologous systems. Signaling through these heterodimers is predominantly driven by the 5-HT2C protomer. Notably, in the 5-HT2A-5-HT2C and 5-HT2B-5-HT2C heterodimers containing the 5-HT2C, the binding of ligands selective for the 5-HT2A or 5-HT2B protomers was suppressed, even though these receptor subtypes were correctly expressed on the cell surface. Conversely, selective 5-HT2A or 5-HT2B antagonists were ineffective in blocking 5-HT signaling in the presence of the 5-HT2C protomer, whereas antagonists of the 5-HT2C protomer entirely inhibited 5-HT signaling in 5-HT2A-5-HT2C and 5-HT2B-5-HT2C heterodimers. In contrast, signaling in 5-HT2A-5-HT2B heterodimers can be blocked by either selective 5-HT2A or 5-HT2B antagonists. Through the study of dimerization involving the 5-HT2A and 5-HT2B receptors with a defective 5-HT2CDCter receptor (capable of binding 5-HT but unable to activate signaling pathways leading to inositol phosphate production), it was determined that co-expression of 5-HT2CDCter with 5-HT2A or 5-HT2B protomers eliminates 5-HT-dependent inositol phosphate accumulation by the 5-HT2A5-HT2CDCter and 5-HT2B-5-HT2CDCter heterodimers. Furthermore, the co-expression of 5-HT2C with the defective 5-HT2BDCter 5-HT2B receptor had no effect on 5-HT2C signaling. This observation is attributed to the dominant negative influence of the 5-HT2C protomer on ligand binding and the binding capacity of the other partner [19,189].

Heterodimers formed between the Gi/o-coupled 5-HT1A receptor and the Gq-coupled 5-HT2A receptor have been demonstrated in co-transfected cells. In vivo clusters of 5-HT1A-5-HT2A receptors were identified in the pyramidal cells of the CA1-CA3 regions of the rat hippocampus [195]. These clusters exhibited alterations in response to a forced swim test used as a model for depression. In this model, the clusters decreased in the cerebral cortex of mice but increased under the influence of the 5-HT2A receptor antagonist and low doses of the antipsychotic clozapine [179]. Similar to the interactions observed in the 5-HT2A-5-HT2C and 5-HT2B-5-HT2C heterodimers, the signal transduction mediated by 5-HT1A-5-HT2A heterodimers does not simply summate the signals of each protomer. Radioligand analysis revealed that the 5-HT2A receptor agonist TCB2 substantially reduced the binding of the 5-HT1A receptor agonist ipsapirone in frontal cortical membranes. This finding suggests the presence of allosteric inhibitory interactions within 5-HT1A-5-HT2A heterodimers, where the 5-HT2A protomer exerts a dominant influence over the 5-HT1A protomer, as activation of the 5-HT2A protomer diminishes the affinity of the 5-HT1A protomer for its ligands [195].

In summary, the research findings indicate that 5-HT receptor heterodimers can display asymmetric binding, where one protomer exerts a dominant influence over the other. Furthermore, the resultant connection within these heterodimers may deviate from the intrinsic signaling patterns of each protomer. Consequently, variations in the relative expression levels of distinct 5-HT receptor subtypes within the same cells can profoundly impact the signaling and pharmacological properties of these receptors.

### 3.3. Heterodimerization of Serotonin Receptors with other GPCRs, and with Proteins Other Than GPCRs

It is noteworthy that 5-HT receptors have the capacity to form heterodimers with receptors from other receptor families. Heterodimerization has been observed between 5-HT1A and μ-opioid [20] as well as dopamine D2 receptors [186]. Additionally, heterodimers involving 5-HT2B with angiotensin AT1 receptors [196] and β2-adrenergic (β2-AR) [197] receptors have been documented. Furthermore, 5-HT2A receptors have been found to heterodimerize with metabotropic glutamate mGlu2 receptors [22], D2 receptors [21], and CB1 cannabinoid receptors [23]. Similarly, 5-HT2C receptors have been reported to heterodimerize with MT2 melatonin receptors [198], 5-HT2A receptors, and TrkB receptors [24]. Several of these heterodimers within this extensive list have demonstrated associations with depressive and anxious behavior.

**Figure 5:**
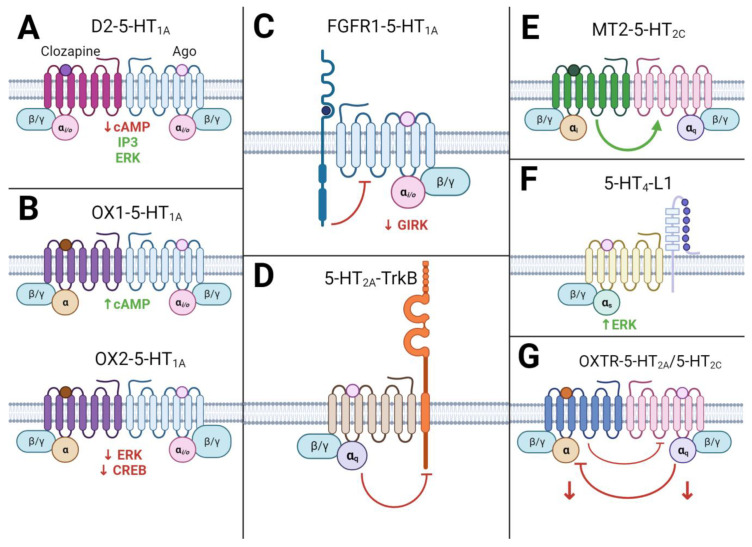
Heterodimerization of 5-HTR with other receptors and proteins. (**A**) Heterodimerization of D2-5-HT1A in the presence of the antipsychotic clozapine and the HT1A agonist significantly inhibits cAMP production, compared with the effect of this combination of drugs on the receptors separately. In addition, production of IP3 and activation of ERK are noted, which occurs only in the case of the heterodimer. (**B**) Heterodimerization of the serotonin HT1A receptor and the orexin OX1 receptor increases cAMP production compared with the HT1A receptor that does not form a heterodimer. Heterodimerization of HT1A and OX2 reduces the level of phosphorylation of ERK and CREB. (**C**) In the 5HT1A-FGFR1 heterodimer, the agonist-activated FGFR1 protomer has an inhibitory effect on the ability of the 5HT1A protomer to open GIRK channels. (**D**) Heterodimerization of 5-HT2A and the BDNF receptor TrkB suppresses basal autophosphorylation of TrkB and prevents agonist-mediated activation of TrkB without affecting 5-HT2A receptor functions. (**E**) When melatonin activates the melatonin receptor MT2 in the 5-HT2C-MT2 heterodimer, unidirectional transactivation of the 5-HT2C protomer occurs. (**F**) 5-HT4-L1 heterodimer exhibits more efficient ERK kinase phosphorylation when stimulated with a 5-HT4 protomer agonist compared to stimulation of the 5-HT4 receptor alone. However, L1 itself does not modulate ERK activation. (**G**) OXTR-5-HT2A and OXTR-5-HT2C heteroreceptors exhibit bidirectional antagonistic interactions. In this case, the 5-HT2C/5-HT2A protomer is dominant and significantly reduces signal transmission from oxytocin receptors. Lite purple dots—serotonin or any selective agonist of corresponding serotonin receptor; purple dot—clozapine; brown dots—agonists of OX1/OX2 receptors; dark blue dot—agonist of FGFR1; dark green dot—melatonin or any MT2 agonist; orange dot—agonist of OXTR; ↑—activation or increase releasing; ↓—inhibition or decrease releasing. Figure was created with biorender.com.

Dopamine D2 and serotonin 5-HT1A receptors play pivotal roles in neurotransmission and are implicated in various human psychiatric disorders. Both dopamine D2 and serotonin 5-HT1A receptors signal through Gi/o, inhibiting adenylate cyclase and negatively modulating cAMP production when expressed individually. Notably, in vivo experiments using proximity ligation assays (PLA) have confirmed the existence of D2-5-HT1A heterodimers in the prefrontal cortex of mice [179]. When cells co-expressing D2 and 5-HT1A receptors were exposed to a single low dose of clozapine (an antipsychotic) along with the 5-HT1A receptor agonist 8-OH-DPAT, there was a substantial reduction in cAMP production compared to the effect of this drug combination on cells expressing only one of these receptors. Co-transfected cells, when stimulated with clozapine and 8-OH-DPAT, exhibited inositol phosphate production and ERK activation. However, these drugs had no impact on cells expressing only one of these receptors, indicating the involvement of novel binding pathways (Figure 5) [186].

This phenomenon suggests that the activation of distinct second messenger pathways depends on whether D2 and 5-HT1A receptors are expressed separately or in conjunction. Activation of 8-OH-DPAT 5-HT1A receptors together with the blockade of the D2 receptor by clozapine can induce conformational changes in the D2-5-HT1A heterodimer, allowing specific recruitment and activation of the Gq protein [186]. It is important to note that the D2-5-HT1A complex possesses unique functional characteristics compared to their respective homodimers. Furthermore, clozapine has been demonstrated to increase the levels of D2-5-HT1A heterodimers in the prefrontal cortex of mice, while the typical antipsychotic haloperidol, a high-affinity D2 antagonist, reduced their presence [179]. These findings collectively suggest that D2-5-HT1A heterodimers activate specific signaling pathways involving inositol phosphate production and ERK activation, and that the levels of these heterodimers can be differentially regulated by various antipsychotic agents.

Moreover, 5-HT1AR has been observed to constitutively form heterodimers with orexin receptors OX1R, which give rise to novel G-protein-dependent signaling. Wang et al. demonstrated that the cellular cAMP expression levels significantly increase in HEK293T cells transfected with both receptors as opposed to the 5-HT1AR group alone. Additionally, 5-HT1AR and OX2R heterodimers downregulate the phosphorylation levels of extracellular signal-regulated kinase (ERK) and cAMP element-binding protein (CREB) [199]. Moreover, in vivo experiments have revealed an association between increased expression of 5-HT1AR/OX1R and depressive behavior in rats [200]. The structurally active 5-HT1AR/OX1R dimer undergoes a transformation from the TM4/TM5 configuration in its basal state to TM6 in the active conformation. Injection of TM4/TM5 peptides into rats subjected to chronic unpredictable mild stress ameliorated their depressive-like emotional state and reduced the number of endogenous 5-HT1AR/OX1R heterodimers in the brain. These findings suggest that 5-HT1AR/OX1R heterodimers are implicated in the pathological processes underlying depression [200].

In recent years, the discovery of the heteroreceptor complex 5HT1AR-FGFR1 has gained attention [201]. This heteroreceptor complex plays a role in regulating neuroplasticity in the hippocampus and serotonergic raphe neurons of rats. This regulation primarily occurs through the allosteric 5HT1A protomer, which enhances the signaling of the FGFR1 protomer, resulting in antidepressant-like effects [202]. Activation of the 5HT1AR-FGFR1 heteroreceptor complex in the raphe nuclei and hippocampus of Sprague Dawley rats using specific agonists has been shown to reduce the ability of the 5HT1AR protomer to open GIRK channels. This reduction is due to an allosteric inhibitory interaction caused by the activation of the FGFR1 protomer, ultimately leading to increased neuronal activity. Notably, in rats with a genetic model of depression, such as the Flinders Sensitive Line, the inhibitory allosteric effect of the FGFR1 agonist on the 5HT1AR protomer failed to impact the GIRK channels in the raphe nuclei but did affect the CA1 region of the hippocampus. A study by Borroto-Escuela et al. has shown significant reductions in immobility time and an increase in swim time in the forced swim test with combined treatment using FGF2 and 8-OH-DPAT, suggesting a potential antidepressant effect of this combination. Conversely, treatment with FGF2 alone led to a small but significant decrease in immobility and an increase in swim time compared to the control group. This underscores the possibility that co-activation of protomers may contribute to more effective antidepressant outcomes [203]. These findings provide new insights into the pathogenesis of depressive disorders [204].

Research exploring the interaction between oxytocin and serotonin receptors shows promise. Oxytocin is implicated in modulating social and cognitive behaviors like attachment, reward, motivation, and responses to fear, anxiety, and stress [205]. There’s a substantial degree of colocalization of serotonergic and oxytocinergic nerve terminal processes, as well as 5-HT2A/C receptors and oxytocin receptor systems in cortical and subcortical forebrain regions [206]. Oxytocin may exert anxiolytic effects through oxytocin receptors (OXTRs) expressed in serotonin neurons [207]. Moreover, the formation of heteroreceptor complexes involving oxytocin receptors with 5-HT2AR and 5-HT2CR has been demonstrated [208,209].

The dynamic assembly of heteroreceptor complexes OXTR-5-HT2AR, OXTR-5-HT2CR, and OXTR-D2R, particularly within the limbic system and nucleus accumbens, plays a pivotal role in modulating social and cognitive behavior. These complexes exhibit bidirectional antagonistic allosteric receptor–receptor interactions, particularly in the OXTR-5-HT2AR and OXTR-5-HT2CR heterocomplexes. Notably, both the 5-HT2AR and 5-HT2CR protomers exert significant influence and, upon activation, substantially attenuate signaling by the OXTR protomer in an in vitro model [208,209]. The functional significance of the heteroreceptor complex in vivo was validated through the intraperitoneal administration of the selective 5-HT2CR antagonist SB242084 to mice, followed by intraperitoneal injection of oxytocin. This co-treatment significantly augmented the effect of OT-mediated hypolocomotion compared to oxytocin administration alone, underscoring the synergistic impact of the drug combination. Interestingly, the separate administration of SB242084 exhibited a contrary effect, leading to an increase in motor activity; it suggests that this is not simply an additive effect of the drugs [208].

The interaction between melatonin MT2 receptors and serotonin 5-HT2C receptors is of interest. Stimulation of the 5-HT2C/MT2 heterodimer by melatonin not only activates the Gi/cAMP pathway typical for MT2 but also transactivates the Gq/PLC pathway of the 5-HT2C protomer. Importantly, this transactivation is unidirectional and is not observed for the MT2 protomer upon stimulation with 5-HT [210]. It is worth noting that the antidepressant agomelatine, which functions as both an MT2 receptor agonist and a neutral 5-HT2C receptor antagonist, interacts with this heterodimer. Agomelatine exerts synergistic effects on both protomers in the dimer, producing a more potent effect compared to using 5-HT2C antagonists and MT2 agonists separately. For instance, acute administration of agomelatine in rats results in an increased level of BDNF mRNA in the prefrontal cortex compared to the administration of melatonin and the 5-HT2C receptor antagonist S22153 separately [198,211,212,213]. In vivo studies have demonstrated the antidepressant effect of agomelatine across various depression models, including the forced swim test, the learned helplessness test, and the sucrose preference test. Moreover, it was observed that the treatment of animals with agomelatine had a more potent antidepressant effect in a learned helplessness test compared to the separate administration of melatonin agonists and 5-HT2C receptor antagonists [198].

Numerous studies have reported the formation of heterodimers involving non-GPCR proteins. For example, 5-HT receptors, including the 5-HT4 and 5-HT7 subtypes, have the capacity to form heterodimers with the adhesion molecule L1 [214], CDK5 [215], or CD44 [163]. Additionally, serotonin receptors 5-HT2A have been reported to form heterodimers with the TrkB protein [24].

The presence of 5-HT4/L1 heterodimers was identified in the hippocampus and cerebral cortex of mice through immunofluorescence microscopy and co-immunoprecipitation. This indicates the existence of physical interactions between the molecules in vivo [214]. The 5-HT4/L1 heterodimer demonstrates increased efficiency in ERK kinase phosphorylation when stimulated with the 5-HT4 receptor agonist BIMU8, compared to the stimulation of the 5-HT4 receptor in isolation. Notably, the L1 adhesion molecule itself does not have a modulating effect on ERK activation. These findings indicate that heterodimerization between the 5-HT4 receptor and the L1 adhesion molecule facilitates ERK activation through the 5-HT4 receptor [214].

The research conducted by Ilchibaeva et al. sheds light on novel molecular mechanisms governing the interaction between the serotonin system and the neurotrophic factor BDNF. Additionally, co-immunoprecipitation and immunofluorescence analyses demonstrated the colocalization of 5-HT2AR and TrkB in the frontal cortex, hippocampus, and striatum of mice. This finding confirms the formation of protein complexes in vivo. Heterodimerization of 5-HT2A-TrkB has the remarkable effect of suppressing basal TrkB autophosphorylation and preventing agonist-mediated TrkB activation, all without affecting 5-HT2A receptor function. Importantly, these inhibitory effects on TrkB function can be reversed through pharmacological blockade of the 5-HT2A receptor. Additionally, the stoichiometry of heterodimerization has been shown to play a significant role. It is plausible to assume that changes in the relative expression of both receptors and the corresponding alterations in the rate of heterodimerization may constitute a mechanism for the specific regulation of TrkB functions within different brain regions under normal and various pathological conditions [24].

## 4. Conclusions

The pathogenesis of depression is undeniably influenced not only by the serotonergic system but also by the diversity of serotonin receptors and the intricate molecular mechanisms activated by serotonin. This complexity offers extensive opportunities for further exploration of the serotonin system’s involvement in mood disorders. Moreover, it provides a foundation for the development of next-generation antidepressants with improved efficacy and faster therapeutic responses.

Allosteric receptor–receptor interactions within heteroreceptor complexes can diversify and specify receptor responses. These interactions operate through conformational changes induced allosterically in distinct domains, subsequently altering the function and pharmacology of receptor protomers. In recent years, there has been a growing interest in allosteric receptor–receptor interactions within receptor complexes, both in the fields of biology and medicine. They are seen as promising targets for the treatment of neurological and psychiatric disorders [216]. The exploration of bivalent compounds with selectivity for specific dimers holds promise. Divalent ligands often exhibit increased binding affinity and selectivity compared to their monomeric counterparts, and they can induce effects distinct from those caused by monomeric ligands. It is essential to highlight that the development of such bivalent ligands is primarily crucial for investigating the physiological properties of dimeric receptor complexes in vivo, a task that currently presents challenges. This approach could potentially pave the way for a novel class of antidepressants [217]. Another intriguing avenue of research involves the creation of ligands designed to either stabilize specific “beneficial” dimers or disrupt the formation of “harmful” dimers [218]. Additionally, a straightforward strategy would be the development of combination drugs containing ligands that target different receptors or ligands that act on both protomers within a dimer, akin to the mechanism of action of agomelatine. The connection between synaptic transmission dysadaptation and imbalanced or dysfunctional expression of heteroreceptor complexes has been well-documented in various diseases, including depression [219].

The existing knowledge about the role of oligomeric complexes formed by 5-HT receptors in the brain indicates that these complexes serve as molecular hubs for the dynamic integration and adaptation of biological signals. These include the regulation of neurotransmitter balances, synaptic plasticity, and the modulation of behavioral and emotional responses. The imbalance between monomeric and oligomeric serotonin receptors likely plays a significant role in the pathophysiological processes leading to depressive disorders. Consequently, restoring these integrative molecular mechanisms holds promise for producing antidepressant effects and represents a compelling target in the development of new drugs for depression treatment. Such drugs might also ameliorate the delayed antidepressant effects associated with SSRIs and other existing antidepressants.

## Figures and Tables

**Table 1:** Classification of serotonin receptors [18,28,29,30,31,32,33,34,35,36,37,38,39,40,41,42,43].

Receptor Family	Subtype	Location	Effectors	Mechanism	Function in the CNS
5-HT1	5-HT1A	CNS (pituitary gland, rostral raphe nuclei, hippocampus, prefrontal cerebellar cortex, basal ganglia, amygdala, globus pallidus, putamen, caudate nucleus), vessels	Gi/o Gβγ	↓cAMP ↑ GIRK, ↑ VGCC Non-canonical: ↑ PLC, ERK, ↑small G-proteins (RhoA)	membrane hyperpolarization, reduced neuronal excitability and frequency of nerve impulse generation, aggression, anxiety, addiction, appetite, memory, mood, nociception, sleep, thermoregulation
	5-HT1B				aggression, anxiety, learning, addiction, memory, mood
	5-HT1D				nociception, anxiety
	5-HT1E				insufficiently studied—memory?
	5-HT1F				anxiety, nociception
5-HT2	5-HT2A	CNS (cerebral cortex, basal ganglia, amygdala, choroid plexus, hypothalamus, hippocampus, caudate nucleus, putamen, globus pallidus, substantia nigra), PNS, platelets, blood vessels, smooth muscle	Gq/11	↑ activity of PLC, ↑ Non-canonical: ↑ PLA2, ↑ PLD, ↑ Src/Act, ↑ ERK	anxiety, appetite, addiction, cognition, imagination, learning, memory, perception, sleep, thermoregulation, motivation
	5-HT2B				anxiety, appetite, sleep
	5-HT2C				anxiety, appetite, addiction, mood, sleep, thermoregulation
5-HT3	5-HT3A	CNS (area postrema, solitary tract, limbic system, hippocampus, cerebral cortex), PNS, gastrointestinal tract	Ligand-gated Na^+^\K^+^ ion channels	Membrane depolarization	involvement in anxiolytic action and cognitive functions, such as learning, attention, memory and fear extinction gag reflex, brain–gut signaling circuitry nausea and vomiting tissue injury-induced pain
	5-HT3B				
5-HT4	5-HT4	CNS (prefrontal cortex, caudate nucleus, putamen, globus pallidus, hippocampus, substantia nigra), PNS, heart, intestines, adrenal glands, bladder, cells of the immune system	Gs	↑ cAMP, Non-canonical: ↑ Src, ERK	synaptic plasticity modulation the release of various neurotransmitters (GABA, acetylcholine, dopamine, histamine, 5-HT) appetite, learning, memory, mood
5-HT5	5-HT5A	CNS (cerebral cortex, amygdala, cerebellum, hypothalamus, hippocampus)	Gi/o	↓ cAMP	sleep, learning, memory, mood
	5-HT5B		Gi/o	unidentified	unidentified
5-HT6	5-HT6	CNS (dentate gyrus, hippocampus, olfactory tubercle, nucleus accumbens, amygdala, cerebellum)	Gs	↑ cAMP Non-canonical: ↑ ERK, ↑ mTOR, ↑ Cdk5	anxiety, cognition, learning, memory, mood
5-HT7	5-HT7	CNS (thalamus, hippocampus, amygdala, cerebral cortex), gastrointestinal tract, blood vessels	Gs	↑ cAMP Non-canonical: ↑ PKA, MAPK, ERK, stimulation of Rho GTPases (Cdc42 and RhoA) modulation of the Ca^2+^/calmodulin pathway	anxiety, memory, mood, sleep, thermoregulation cytoskeletal reorganization (morphological changes in astrocytes, fibroblasts and neurons)

↑—activation or increase releasing; ↓—inhibition or decrease releasing. CNS—central nervous system; cAMP—cyclic adenosine monophosphate; GIRK—G-protein-gated inwardly rectifying potassium (GIRK) channels; VGCC—voltage-gated calcium channel; PLC—phospholipase C; ERK—extracellular signal-regulated kinase; RhoA—small G-proteins; PLA2—phospholipase A2; PLD—phospholipase D; Src—non-receptor tyrosine kinases; Act—RAC-alpha serine/threonine-protein kinase; mTOR—mammalian target of rapamycin; Cdk5—cyclin-dependent kinase 5; PKA—protein kinase A, MAPK—mitogen-activated protein kinase; Cdc42—small Rho-family guanosine triphosphatase Cdc42.

## Data Availability

No new data were created or analyzed in this study. Data sharing is not applicable to this article.
